# Thermal and Mechanical Properties of Cementitious Waste-Coated Polyamide 6 Composites

**DOI:** 10.3390/polym18161947

**Published:** 2026-08-09

**Authors:** Ayfer Donmez Cavdar, Husnu Yel, Sevda Boran Torun, Murat Ertas, Salim Hiziroglu

**Affiliations:** 1 Department of Forest Industry Engineering, Faculty of Forestry, Karadeniz Technical University, 61080 Trabzon, Türkiye; adonmez@ktu.edu.tr; 2 Department of Forest Industry Engineering, Faculty of Forestry, Artvin Coruh University, 08000 Artvin, Türkiye; yel33@artvin.edu.tr; 3 Department of Materials and Material Processing Technologies, Karadeniz Technical University, 61900 Arsin, Türkiye; sboran@ktu.edu.tr; 4 Department of Forest Industry Engineering, Faculty of Forestry, Bursa Technical University, 16310 Bursa, Türkiye; murat.ertas@btu.edu.tr; 5 Department of Natural Resource Ecology and Management, Oklahoma State University, Stillwater, OK 74075, USA

**Keywords:** mineral additives, polyamide 6, thermal properties, mechanical properties, hybrid composites

## Abstract

The study investigates pozzolanic materials, silica fume, and fly ash as reinforcement agents in a polyamide 6 (PA6) matrix with cement. The materials were combined in various proportions and applied via ultrasonic coating before being extruded into composite filament samples. Different characterization techniques, including thermogravimetric analysis (TGA) and Differential Scanning Calorimetry (DSC), were employed for evaluation of the samples. Results showed that PA6 composites with silica fume had the highest modulus of elasticity (MOE) but relatively low modulus of rupture (MOR) values for the samples. The addition of cement and fly ash enhanced their overall mechanical properties, increasing MOR and MOE by 24.4% and 81.5%, respectively. TGA indicated improved thermal stability with silica fume, while fly ash displayed the highest residual mass. Neat PA6 maintained a high maximum decomposition temperature (Tmax) but minimal residue. DSC results suggested that while melting temperature remained largely unchanged, cement reduced enthalpy change, indicating limited crystallization. Neat PA6’s low heat deflection temperature (HDT) due to amorphous content led to reduced mechanical strength at high temperatures. Cement enhanced the deformation resistance of the samples, while silica fume provided limited reinforcement. Having fly ash in the samples significantly improved their HDT, thermal stability, and rigidity. Based on the findings in this study, pozzolanic materials demonstrated their potential use as effective fillers for PA6 composites in various applications.

## 1. Introduction

Plastic materials have many advantages for the automotive industry, including the ability to meet complex engineering demands such as aesthetics, safety, comfort, and fuel efficiency, as well as compatibility with electronic components in a cost-effective approach and ease of recycling [1,2]. However, the performance of the additive-free, non-modified polymers is somewhat limited in many applications [3] due to the soft surface, low scratch resistance and limited thermal stability. In the last decade, the use of plastics in the automotive industry has grown significantly. Plastic materials make up approximately 10% of the total vehicle weight, while the amount used per car has increased by about 15–16%. Modern vehicles contain almost 50% of all parts by weight and even more by component number of plastics, which underscores their importance in lightweight design and manufacturing efficiency [1,3]. It is a known fact that the overall share of plastics used in vehicles has sustainably increased [4].

Polyamides are one of the most important engineering thermoplastics and are extensively used in a wide range of applications, including automotive parts, electrical and electronic devices, mechanical parts, construction materials, and 3D printing, due to their excellent combination of mechanical strength, toughness, thermal stability, and chemical resistance [5,6]. Recently, polyamide 6-based, also known as nylon 6-based, composites have received much attention, especially in the automotive industry, as high-performance and lightweight alternatives to metal and rubber units in many industrial applications [7]. However, the polyamides have low stiffness and insufficient dimensional stability at elevated temperatures, which limits their applicability where high-performance or load-bearing components are desired [8,9]. The use of inorganic particulate fillers in polymer matrices is an effective way to overcome the inherent limitations of neat polymers and to extend their application scope, leading to significant improvements in mechanical strength, stiffness, dimensional stability, heat deflection temperature, and long-term durability. Furthermore, the partial replacement of expensive polymers with cheap fillers also reduces the overall material cost [10,11,12,13]. The performance of polymer composites filled with particles is highly dependent on the shape, size and dispersion of the filler in the polymer matrix [14].

Due to their chemical stability and flame retardance, pozzolanic materials such as fly ash (FA) and silica fume (SF) have been extensively studied as low-cost and environmentally friendly fillers in polymer composites [8,15,16]. Furthermore, the utilization of these materials in polymer composites promotes sustainable material engineering by enabling the reuse of industrial by-products such as silica fume and fly ash, which would otherwise cause environmental issues [17]. Fly ash is a heterogeneous pozzolanic by-product composed mainly of silica, alumina, and iron oxides that may improve the dimensional stability and heat deflection temperature (HDT) of polymer composites due to a micro filler effect and restriction of polymer chain mobility [14]. However, poor interfacial adhesion arising from low surface activity and particle aggregation often limits the mechanical performance of FA-filled polymer composites [8,18]. Silica fume (SF) is also known as micro silica, a pozzolanic industrial by-product obtained during the manufacture of silicon metal and ferrosilicon alloys in electric arc furnaces [6]. Silica fume is a very fine amorphous SiO_2_, which can improve the crystallinity and stiffness of polymer matrices because of its large surface area and effective nucleation [19]. However, high nanosilica dosage can cause particle agglomeration, which can degrade the mechanical performance of the composite units by reducing their tensile strength and increasing brittleness [20].

Many studies reported that silica- and cement-based fillers can improve the mechanical and thermal properties of polymers by increasing the efficiency of load transfer and limiting the thermal movement of polymer chains [6,21]. However, the majority of the previous studies are focused on direct mixing or blending of mineral powders into the polymer matrix. Surface coating techniques in which mineral particles are applied to polymer granules before compounding have undergone rather limited investigation in the literature. This type of approach can significantly improve the dispersion of fillers and their interfacial adhesion due to uniform coating of fillers and reduction in agglomeration, and better stress transfer during the extrusion or molding process [22].

Although cementitious and pozzolanic additives can be beneficial for the thermal and mechanical performance of polymer composites, the effect of these additives, particularly on the synergistic interactions in hybrid cementitious coatings, has been studied to a lesser extent. In particular, information on the use of cement and pozzolanic additives such as silica fume and fly ash on the performance of PA6 composites is still limited. Therefore, in the present work, a detailed characterization of mineral-coated polyamide 6 (PA6) composites was performed in order to evaluate their potential use in advanced engineering applications, especially in the automotive industry. Unlike conventional approaches based on the direct blending of mineral fillers into polymer matrices, this study applies cement-based, silica fume–cement-based, and fly ash–cement-based coatings to PA6 granules prior to extrusion. This approach combines the performance modification of PA6 with the valorization of low-cost industrial by-products. A controlled comparison was conducted at a constant total inorganic content to clarify the influence of coating composition and the partial replacement of cement with silica fume or fly ash on the mechanical, thermal, and flammability performance of PA6 composites. This coating-based strategy may provide a practical route for developing lightweight polymer composites with tailored performance characteristics. The chemical characteristics of the mineral-coated PA6 granules were examined using FTIR–ATR spectroscopy to evaluate the effects of the coating process. The mechanical properties, namely flexural strength, flexural modulus, tensile strength and tensile modulus of the extruded composite samples were also determined. The thermal properties of the samples were evaluated by thermogravimetric analysis (TGA), Differential Scanning Calorimetry (DSC) and heat deflection temperature (HDT). The flame-retardant behavior of the samples was also determined according to UL 94 standard.

## 2. Materials and Methods

### 2.1. Materials

**Figure 1:**
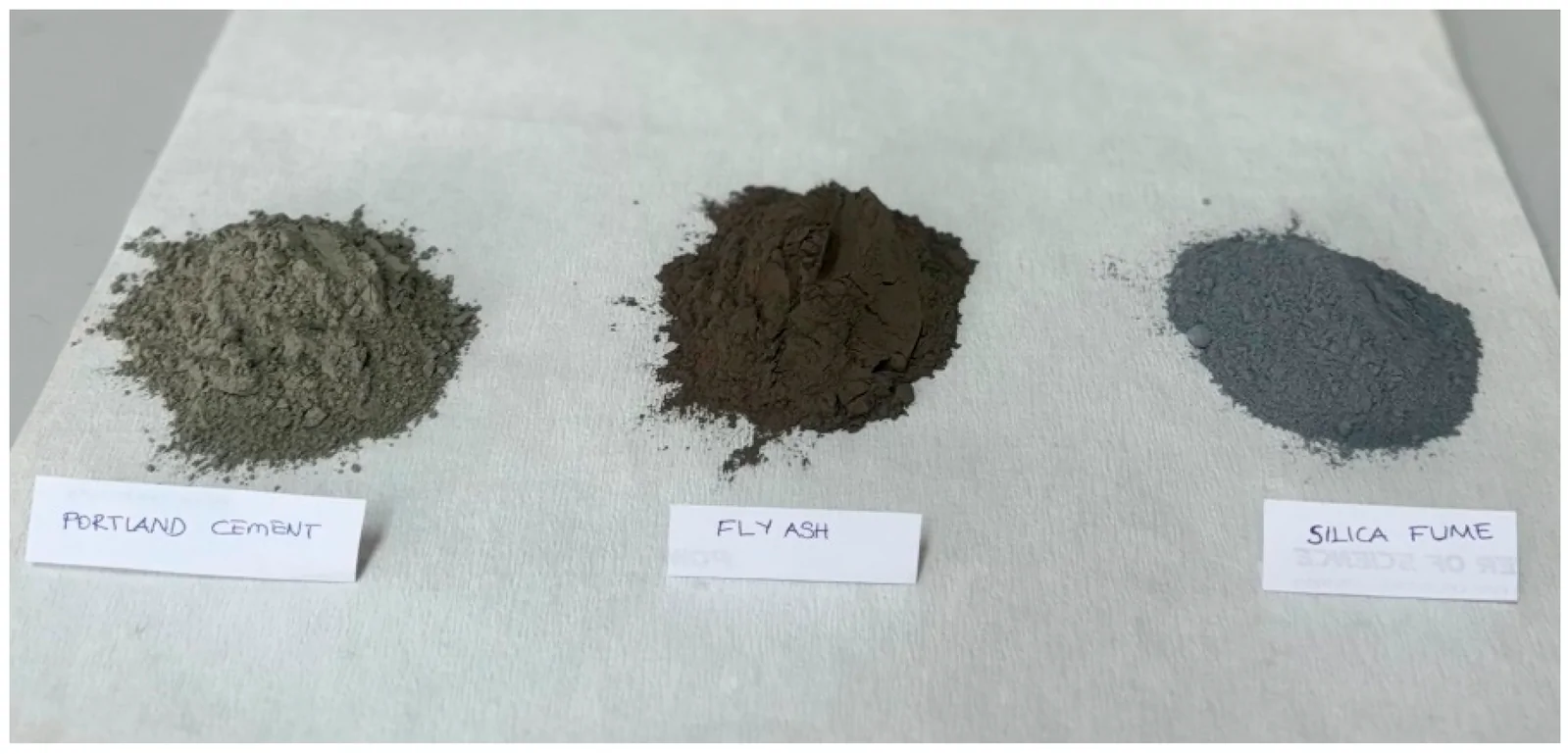
Portland cement, fly ash, and silica fume used for the samples.

Polyamide 6 (PA6) used for the experiments was supplied from Petkim Petrochemical Company, Izmir, Turkey, and employed as the polymer matrix. Portland cement, silica fume, and fly ash as mineral additives were supplied by Askale Cement Company, Trabzon, Türkiye, Etibank Electrometallurgy Plant, Antalya, Türkiye, and ARES Cement Company, Kütahya, Türkiye, respectively. The Portland cement, fly ash, and silica fume used in this research are depicted in Figure 1.

### 2.2. Surface Coating of Polyamide 6 Using Cement, Silica Fume, and Fly Ash

Table 1 summarizes the compositions of the hybrid PA6 composites. The total inorganic content was fixed at 15 wt.%, selected as an intermediate value within the 10–20 wt.% range previously reported to provide favorable mechanical performance in mineral-filled polyamide composites [6,23,24]. The Portland cement-silica fume and Portland cement-fly ash mixtures were separately poured into glass beakers and mixed for 10 min with a magnetic stirrer according to the proportions given in Table 1. In the following step, the cement and cement pozzolanic material mixtures were poured into volumetric flasks, and polyamide 6 granules and 100 g of water were added according to the ratios displayed in Table 1. The mixtures were stirred at a temperature of 50 °C for 30 min using a magnetic stirrer. When the magnetic stirring process was over, the mixtures in the volumetric flasks were submitted to an ultrasonic bath for 30 min to improve the coating of the Polyamide 6 granules with cement and pozzolanic materials. After ultrasonic treatment, the samples were dried in an oven at a temperature of 103 ± 2 °C for 24 h. Then, the excess cement and pozzolanic materials that did not adhere to the surface of polyamide 6 granules were removed by sieving.

### 2.3. Manufacturing of the Cement-Coated PA6 Composite Samples

**Figure 2:**
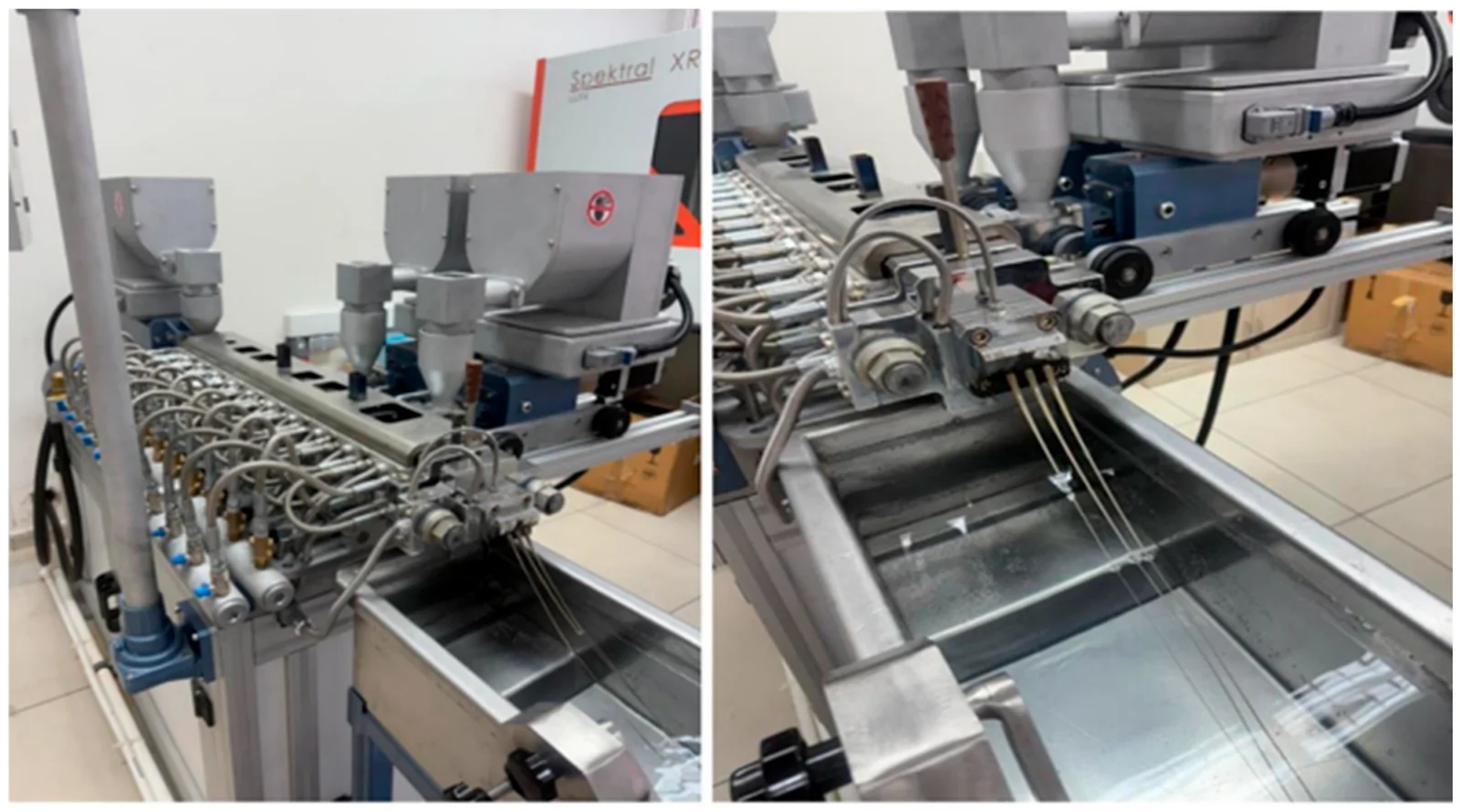
Twin-screw gravimetric extruder.

**Figure 3:**
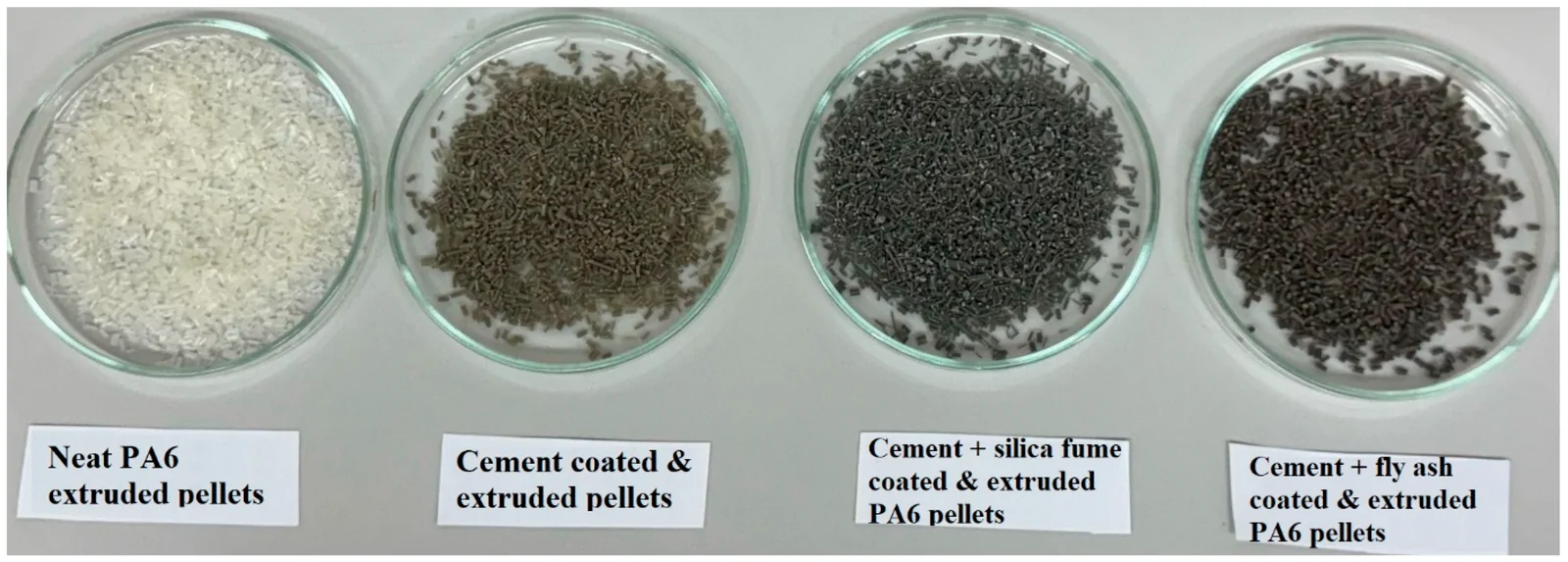
Extruded polyamide 6 filaments coated with cement and pozzolanic materials.

After the drying process, as shown in Figure 2, the Polyamide 6 granules coated with cement and pozzolanic materials were extruded with a twin-screw extruder equipped with dual side feeders. The coated PA6 granules were fed into the extruder, and the compounding process was carried out at a screw speed of 100 rpm, with the temperature zones set between 200 and 240 °C. The hot composite strands exiting the extruder were passed through a cold-water bath for cooling and solidification. The cooled strands were subsequently converted into pellets using a granulator. Figure 3 depicts extruded filaments of the cement-coated PA6 composite. The ground samples were oven-dried to remove any residual moisture before pressing. The dried samples were then hot-pressed at a temperature of 240 °C for 10 min. The samples were then cut into smaller pieces, and their dimensions were measured for different tests.

### 2.4. Test Methods

#### 2.4.1. Fourier Transform Infrared Spectroscopy–Attenuated Total Reflection (FTIR-ATR) Analysis

Prior to extrusion, FTIR–ATR analysis was performed on the oven-dried, mineral-coated PA6 granules using a Shimadzu IRSpirit, Tokyo, Japan, spectrometer equipped with a QATR-S diamond ATR accessory to evaluate the effects of the coating process. The ATR-FTIR spectra of the granules were recorded in the wavenumber range of 650–4000 cm^−1^ and 24 scans at 16 cm^−1^.

#### 2.4.2. Mechanical Tests of the Samples

The mechanical tests of the mineral-coated PA 6 composite samples were performed on a Universal Testing Machine Zwick/Roell Z010, Ulm, Germany, (5–50 kN). The flexural strength and flexural modulus of elasticity of the samples were measured according to the ASTM D790-2004 standard [25], and the tensile strength and tensile modulus of elasticity were measured according to the ASTM D638-1994 standard [26]. A total of 15 specimens of 165  ×  19  ×  3 mm were used for the tensile test, while 12 specimens with the dimensions of 127  ×  12.7  ×  3 mm were prepared for the flexural test.

#### 2.4.3. Thermal Analyses

The thermal stability and residual mass of the samples were investigated using thermogravimetric analysis (TGA) employing a Hitachi Hi-Tech STA7200 thermal analyzer (Hitachi High-Tech America, Inc., Schaumburg, IL, USA). The samples were heated from room temperature up to 1000 °C at a constant heating rate of 10 °C/min in a nitrogen atmosphere with a flow rate of 200 mL/min.

Differential Scanning Calorimetry (DSC) analyses were performed to study the thermal properties of the polyamide 6 granules coated with Portland cement, fly ash, and silica fume, such as glass transition temperature, melting temperature, and crystallization temperature. DSC analysis was performed using a Perkin Elmer (Model DSC 8000), Shelton, CT, USA instrument. The thermogravimetric measurements were performed in the temperature range of 25 °C to 300 °C at a heating rate of 10 °C/min and a nitrogen flow of 20 mL/min. First, samples were heated up to a temperature of 300 °C, and during analysis, they were cooled down and reheated up to 300 °C for testing. The crystallinity degree of the samples was calculated using the following equation [27]:

(1) $$\mathrm{Crystallinity}:\;X_{c}(\%)=\left(\frac{{∆H}_{e}}{{∆H}_{0}\times W}\right)\times 100$$

Δ*H_e_* and Δ*H*_0_ represent the heat fusion (J/g) of the composite and 100% crystalline polyamide 6, respectively. *W* represents the plastic ratio. The Δ*H*_0_ value of polyamide 6 was taken as 230  J/g in this study [28].

Heat deflection temperature (HDT) of samples was determined by using a Ceast HV3, Turin, Italy, instrument following the ASTM D648 standard. The test specimens with dimensions of 127 × 12.7 × 3 mm were loaded with 0.46 MPa and heated at a rate of 120 °C/h up to the deformation onset [29].

The flammability performance of the composites was evaluated by a vertical flame chamber operated according to UL 94 standards [30]. Five samples were tested in the UL 94 vertical fire test. The burning criteria are given in Table 2. Sample dimensions were 120 mm × 12.5 mm × 2 mm. The vertical burning test was used to assess the combustion behavior of the specimens. The specimens were clamped from the top in a vertical position. The samples are classified as V-0, V-1, or V-2 based on their burning behavior after flame application. The V-0 rating indicates the maximum amount of flame retardancy, requiring the shortest after flame time and excluding flaming droplets that ignite the cotton indicator. The V-1 classification permits significantly longer after flame periods while continuing to avoid ignition of the cotton indicator by burning droplets. In contrast, the V-2 classification admits burning drops that may ignite the cotton indicator, indicating less flame-retardant effectiveness than V-0 and V-1. The ignition procedure was divided into two subsequent stages. In the first stage, the bottom end of the specimen was exposed to a flame for 10 s, and the after-flame time was measured as *tV*1 after removal of the ignition source. In the second stage, the specimen was again exposed to the burner immediately after the extinguishing of the first flame for 10 s, and the subsequent after-flame time was recorded as *tV*2. The total after-flame time (*tV*) was calculated as

(2) tV=tV1+tV2

Other physical phenomena, such as the behavior of the melt dripping and the ignition of a cotton indicator placed under the specimen, were carefully observed and recorded during the test.

#### 2.4.4. Statistical Analysis

The mechanical test data, namely tensile and flexural properties of the samples, were statistically analyzed by the software SPSS (version 22.0). The one-way analysis of variance (ANOVA) was used to find out whether there were significant differences between the sample groups. Duncan’s multiple range test at a 5% significance level (*p*  <  0.05) was also used to identify the homogeneous subsets within each group.

## 3. Results and Discussion

### 3.1. FTIR-ATR Characterization

**Figure 4:**
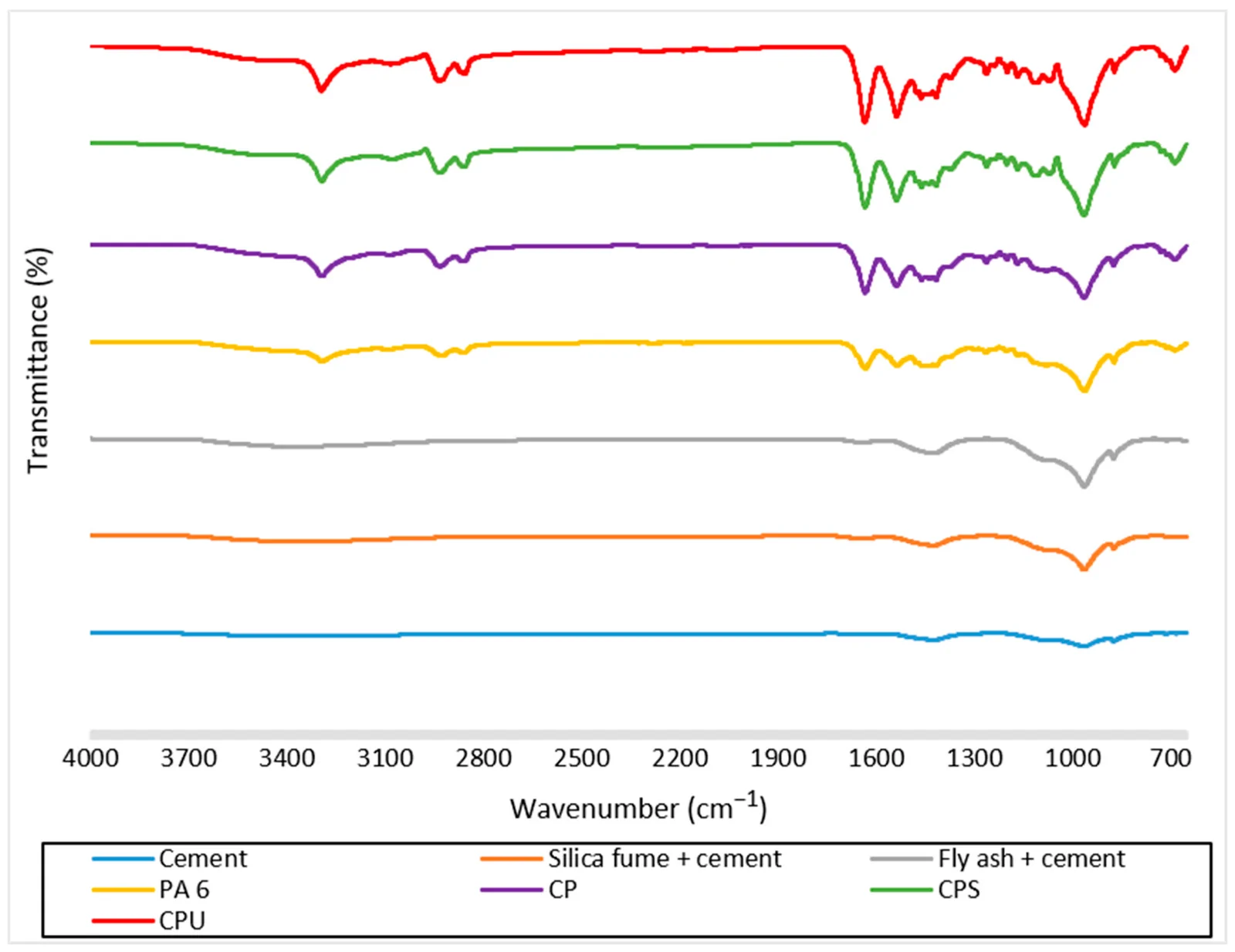
ATR-FTIR spectra of the mineral-coated PA6 granules.

The FTIR–ATR spectra of PA6, CP, CPU, and CPS granules as well as cement, the silica fume–cement and fly ash–cement mixtures are presented in Figure 4. PA6 exhibited its characteristic bands at approximately 3295 cm^−1^, 1635 cm^−1^, and 1535 cm^−1^, assigned to hydrogen-bonded N–H stretching, amide I (C=O), and amide II (N–H and C–N combination) vibrations, respectively [31]. Comparison of the silica fume–cement mixture with the CPS granules showed that the N–H band position remained unchanged, while its intensity, calculated from the raw spectral data, decreased by approximately 15%. Only minor variations were observed in the other characteristic bands and the fingerprint region. The bands associated with cement and the pozzolanic additives, particularly the Si–O-related absorptions in the 900–1100 cm^−1^ region, were also retained in the CPS and CPU spectra [32,33]. The absence of distinct new bands or substantial peak shifts indicates that coating PA6 with cement, silica fume–cement and fly ash–cement mixtures did not cause detectable degradation of the polymer structure, although the limited changes in band intensity may reflect physical and interfacial interactions among the components.

### 3.2. Mechanical Properties

**Figure 5:**
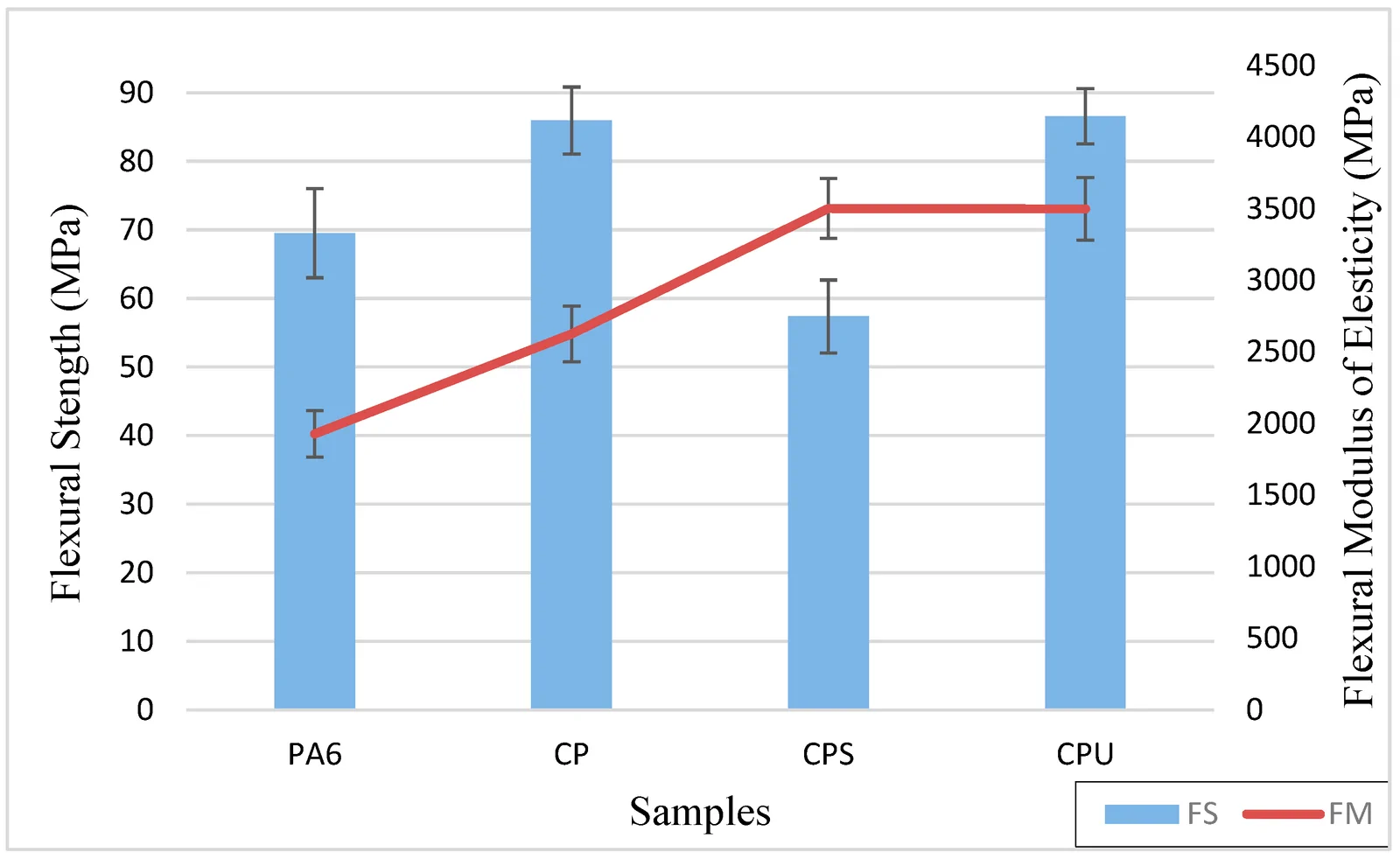
Flexural properties of the mineral-coated PA6 composite samples.

Values of the flexural strength (FS) and flexural modulus of elasticity (FM) of the mineral-coated PA6 composite specimens are illustrated in Figure 5. The addition of cement and cement fly ash mixture to the polymer matrix results in a significant improvement in the values of FS and FM. The cement- and fly ash-coated polyamide 6 composites (CPUs) showed the highest flexural strength, whereas the silica fume- and cement-coated composites (CPSs) showed the lowest value. Fly ash and cement significantly enhanced the flexural strength of polyamide 6 (PA6), while silica fume decreased it. This suggests that the fly ash was well dispersed in the PA 6 matrix, which improved the interfacial adhesion between the polymer and cementitious phases, allowing efficient stress transfer and therefore improving the composite stiffness and the flexural strength [14,21]. This reduction in FS is probably due to the highly reactive and fine nature of silica fume, which gives rise to a more rigid but brittle matrix structure due to its strong interfacial bonding, agglomeration, and limited polymer chain mobility [20,34]. At high contents, the agglomeration of nanosilica reduces the tensile strength and increases the brittleness [20].

The flexural strength results of the samples were opposite to the highest flexural modulus of elasticity (FM) for cement and silica fume (CPS) coated PA6 composites, while the neat PA6 control samples had the lowest value. This enhancement could be due to the crystalline nature of the fillers. The modulus of elasticity increases with increasing crystallinity of the polymer composite phase, since crystalline regions are stiffer than the amorphous regions, which was also confirmed by the DSC results [12,21]. The neat PA6 has low FS and FM values because of the lack of reinforcing fillers, limiting the load-bearing capacity and stiffness under stress [5]. Both silica fume–cement and fly ash–cement mixtures resulted in a greater increase in flexural modulus of PA6 than cement.

**Figure 6:**
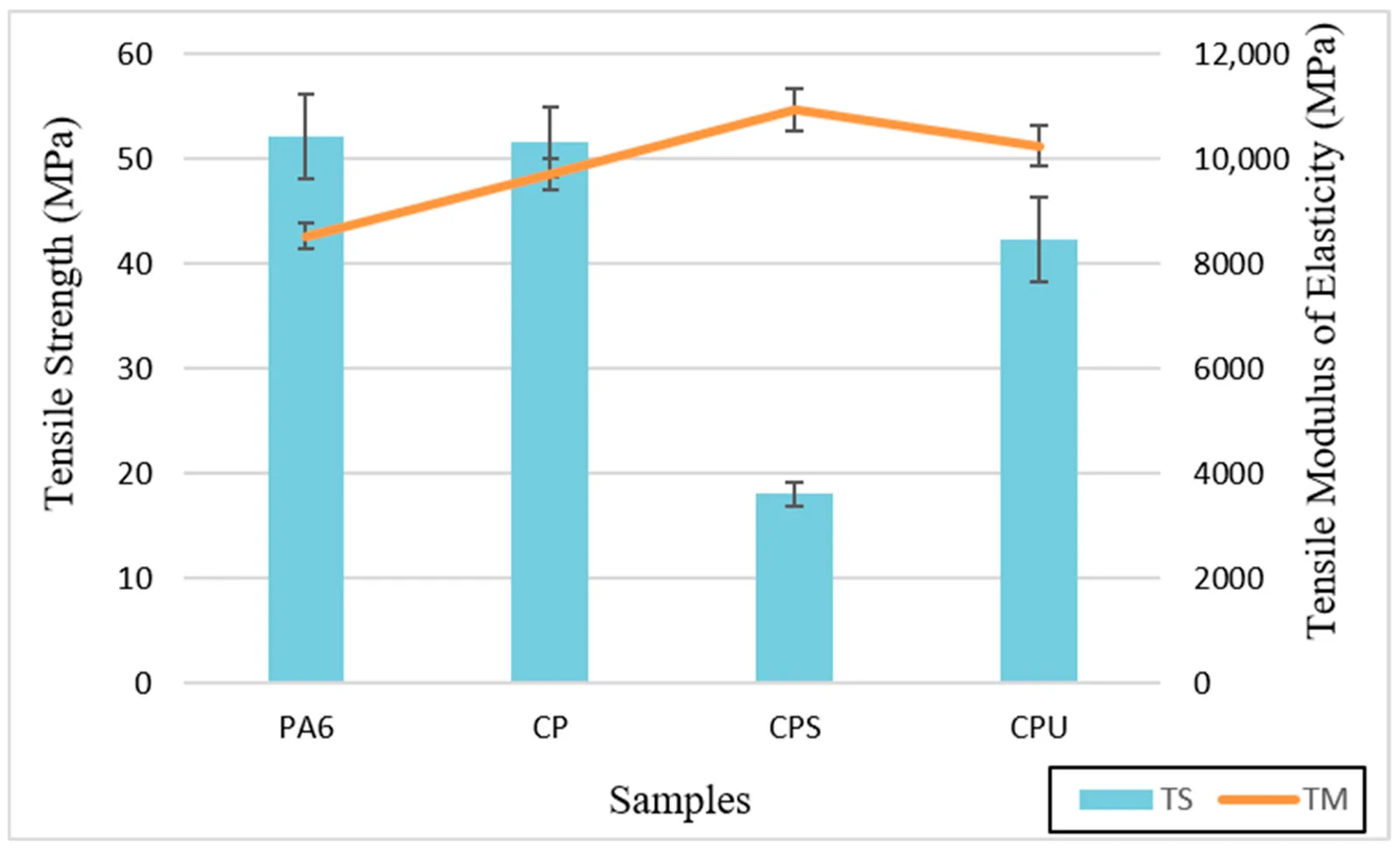
Tensile strength and tensile modulus of elasticity values of the mineral-coated PA6 composite samples.

Figure 6 presents the tensile strength (TS) and the tensile modulus of elasticity (TM) values of the mineral-coated PA6 composites. The results demonstrated that the addition of mineral fillers had different effects on the tensile behaviors of the polyamide 6 matrix compared to the flexural properties. The incorporation of only Portland cement (CP) did not alter the tensile strength (TS) significantly, but it promoted a significant increase in the tensile modulus (TM) that represents lower molecular mobility and increased rigidity due to filler-matrix interactions. The tensile modulus (TM) of the PA6 matrix was significantly improved by the cement–fly ash and cement–silica fume blends, but its tensile strength (TS) decreased at the same time. The decrease in the tensile strength of the PA6 composite is due to the poor interfacial adhesion between the fillers and the polymer matrix, which hinders the effective transfer of stress to the stronger particles of the silica fume and fly ash [14,21]. The results show that the fly ash and silica fume increased the rigidity of the matrix structure of the PA6 composites but were fragile [20]. The results were in agreement with those of previous studies [6,14].

The fly ash–cement mixture was identified as the best mineral coater to improve both strength and rigidity and could be considered as a sustainable pozzolanic coating for polyamide-based composites.

### 3.3. Thermal Properties

#### 3.3.1. Thermogravimetric Analysis (TGA) of the Samples

**Figure 7:**
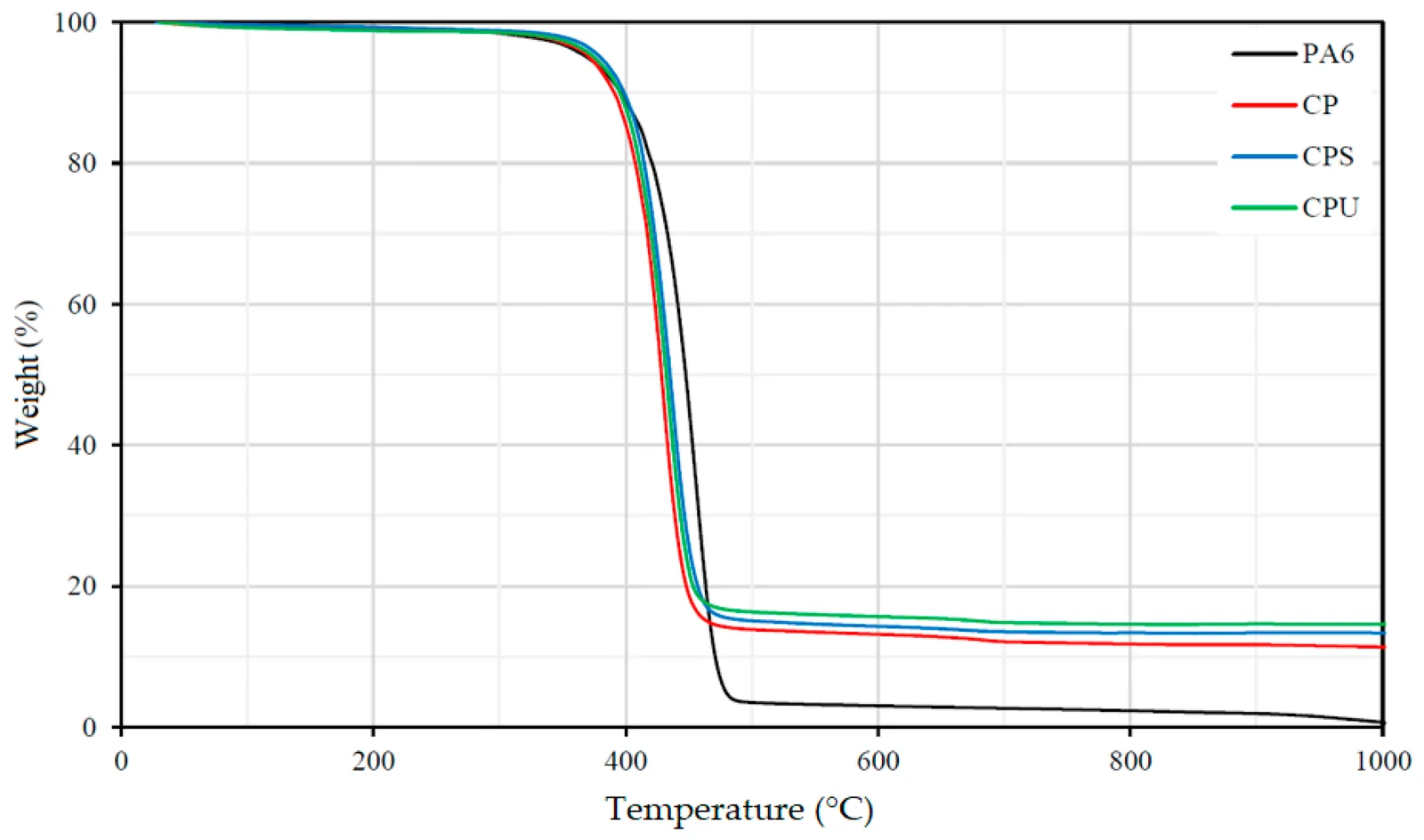
TGA curves of mineral-coated PA6 composites.

**Figure 8:**
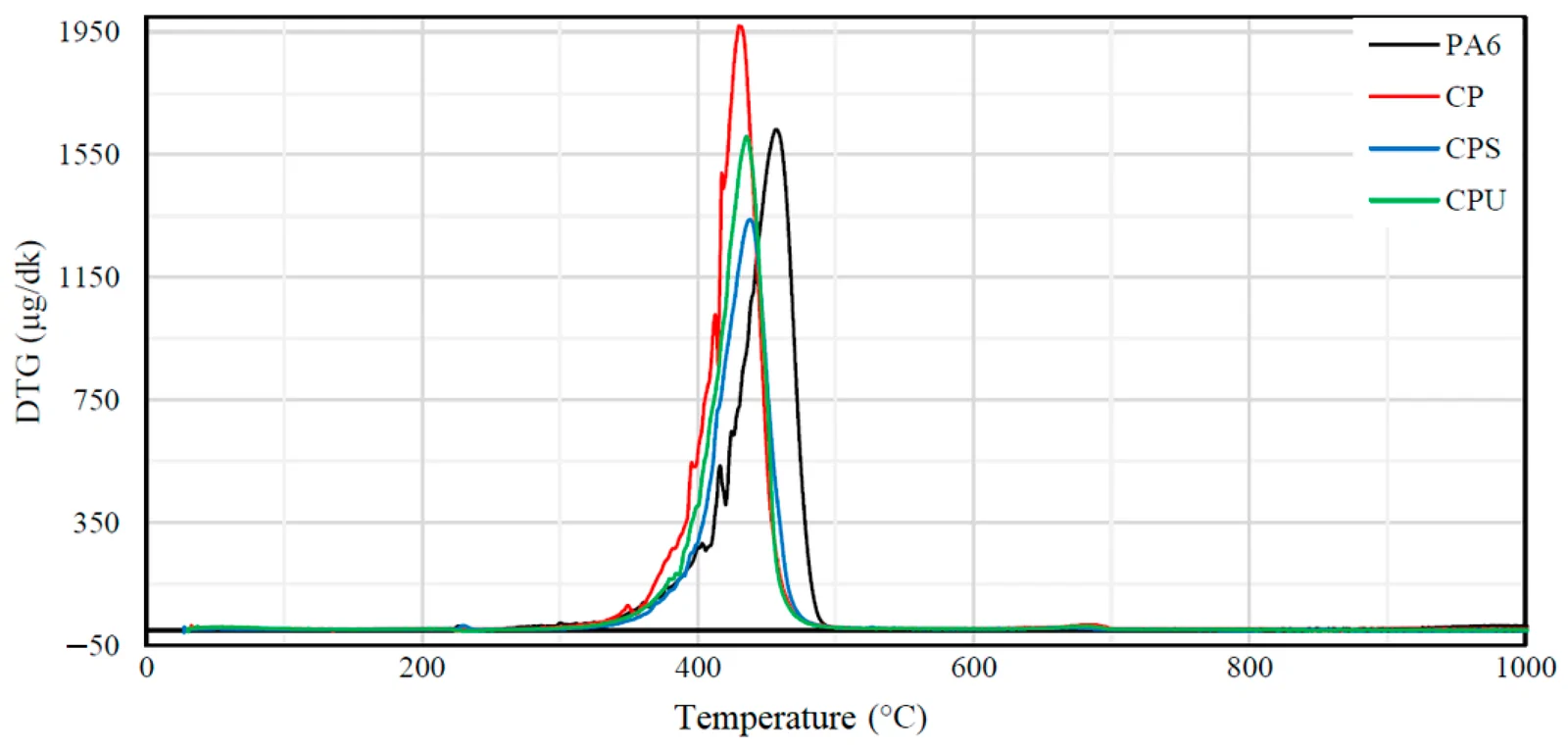
DTG curves of the mineral-coated PA6 composites.

Thermogravimetric analysis (TGA) was used for assessment of the thermal degradation behavior of the composites prepared by the incorporation of different mineral additives such as cement, silica fume, and fly ash into PA6 (polyamide 6). The TGA and DTG plots are shown in Figure 7 and Figure 8, respectively. Table 3 displays the weight loss of the composite samples with temperature and the residual mass at a temperature of 1000 °C.

The PA6 sample lost 5% and 10% of its mass at 369.4 °C and 396.2 °C, respectively, and exhibited the highest decomposition rate at 457.6 °C. The residue mass was very low (0.4%), which indicates that PA6 retains its structure up to a certain temperature and does not leave an inorganic residue after decomposition. Another reason for the relatively high value of Tmax is the thermal stability and thermoplastic nature of the linear PA6 structure [5].

In the CP composite, the combined effect of cement and polyamide was observed. While T%5 and T%10 values were comparable to the neat PA6, Tmax decreased to 431.3 °C, indicating an earlier onset of thermal decomposition. The cement phase in the polymer matrix may have improved heat transfer between polymer chains, leading to degradation [35]. On the other hand, the residual mass increased to 11.3%, which may be attributed to the inorganic components of the cement phase, which remain stable at high temperatures.

The CPS sample with silica fume presented the highest values of T%5 (379.9 °C) and T%10 (398.6 °C) compared to all the systems, which suggests that silica fume improves thermal stability in the composite matrix. In fact, additives based on nanosilica are able to create a barrier effect in the polymer matrix, slowing the diffusion of gases and heat transfer [22]. The Tmax value (438.5 °C) was slightly lower than that of neat PA6 but higher than that of the CP sample. The residual mass was 13.4%, in agreement with the inorganic content of silica fume.

The CPU sample showed moderate thermal resistance with a 5% decomposition temperature of 375.0 °C and 10% decomposition temperature of 395.3 °C. Its Tmax value was 435.7 °C, which was lower than the CPS sample but higher than the CP sample. The most striking result was the maximum residual mass (14.6%) found for the CPU composite. The presence of silicates, aluminates, and other metal oxides in the complex composition of fly ash causes the increase in the residue at high temperatures [17]. Also, the fly ash phase may limit the thermal degradation of the polymer by retarding chain scission, but its contribution to the improvement of Tmax is limited.

The results showed that mineral additives such as cement, silica fume, and fly ash had a significant effect on the degradation behavior of polyamide-based matrices. Silica fume in particular is remarkable for the delaying effect on the onset of degradation, while fly ash is characterized by the higher residual mass. Cement has a small negative effect on thermal stability but increases the retention of residues at high temperature.

#### 3.3.2. Differential Scanning Calorimetry (DSC) Analysis of the Samples

**Figure 9:**
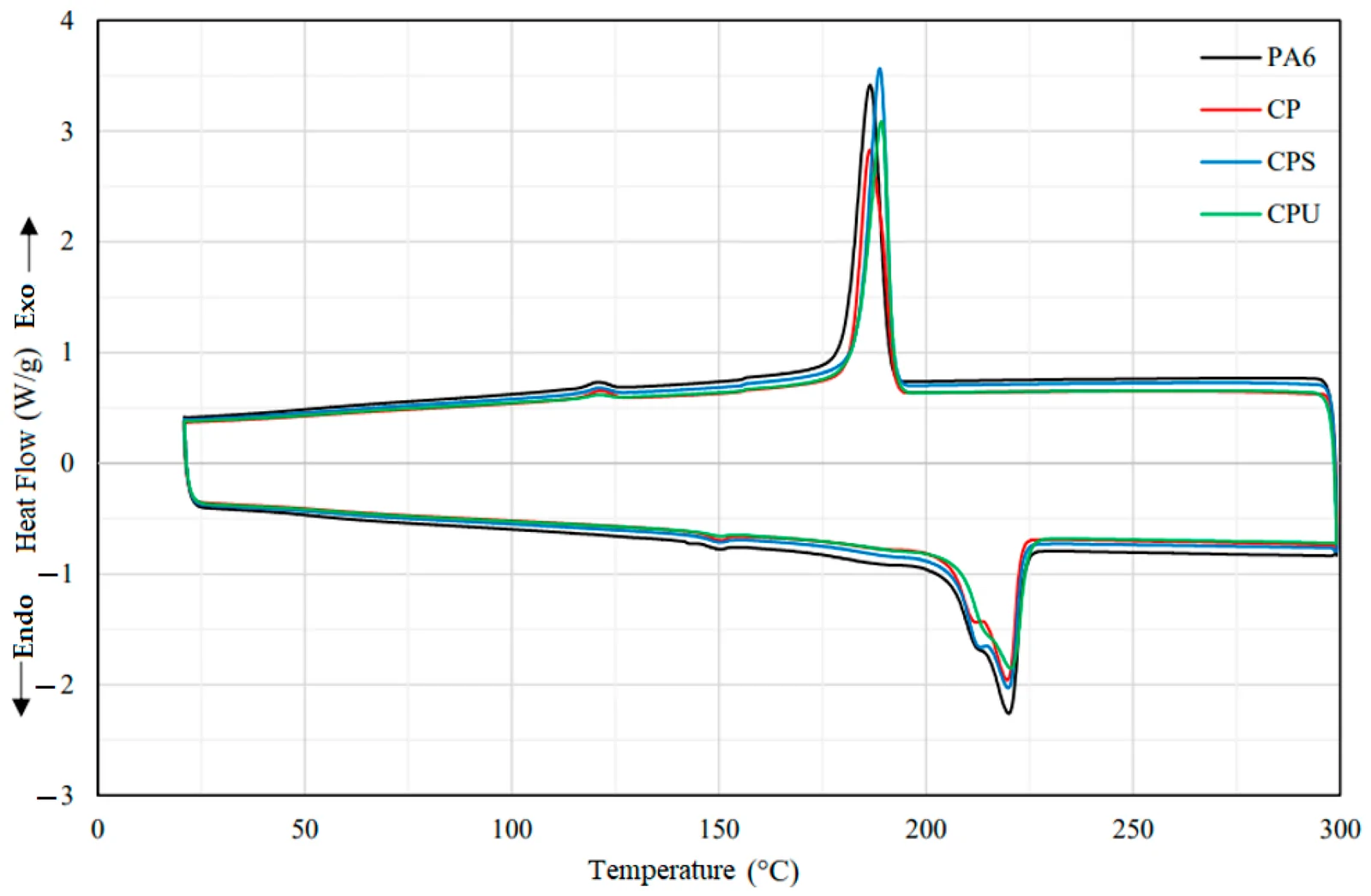
DSC analysis results of the mineral-coated PA6 composites.

Figure 9 and Table 4 show the DSC analysis results of the composite samples. Neat PA6 has a melting temperature (Tm) of 221.6 °C and a melting enthalpy (ΔHm) of 108.6 J/g. The values show that the transition of polyamide to the crystalline phase requires high energy and that it forms a well-organized crystalline structure.

It was found that additives such as cement, silica fume, and fly ash did not significantly affect the melting temperature, as all samples showed similar Tm values. However, the decrease in ΔHm values indicates that these additives disrupt the crystallization order of the polymer chains and inhibit the crystallization phase formation [20]. Specifically, the ΔHm of the cement-coated PA6 (CP) showed a significant decrease (17% reduction), meaning that cement has a stronger inhibition on crystallinity.

The crystallization temperature (Tc) and crystallization enthalpy (ΔHc) of PA6 were 186.6 °C and 119.0 J/g, respectively. Such high values indicate that PA6 rapidly converts to the crystalline phase during cooling. Regarding the addition of silica fume (CPS) and fly ash (CPU), the Tc values were increased, which means that crystallization starts earlier in the cooling stage. Such findings imply that these additives serve as heterogeneous nucleation sites, enhancing crystallization [19]. Interestingly, the crystallization parameters of silica fume (Tc and ΔHc) were closer to those of neat PA6, suggesting that it interferes less with the crystallization process.

The crystalline structure of the polyamide matrix is broken down by cement and pozzolanic additives, resulting in a decrease in melting enthalpy and degree of crystallinity. Of the additives, silica fume was found to give the best results, increasing the crystallization temperature and maintaining crystallinity. While fly ash also increased crystallinity to some extent, it was not as effective as silica fume.

#### 3.3.3. Heat Deflection Temperature (HDT) Analysis of the Samples

**Figure 10:**
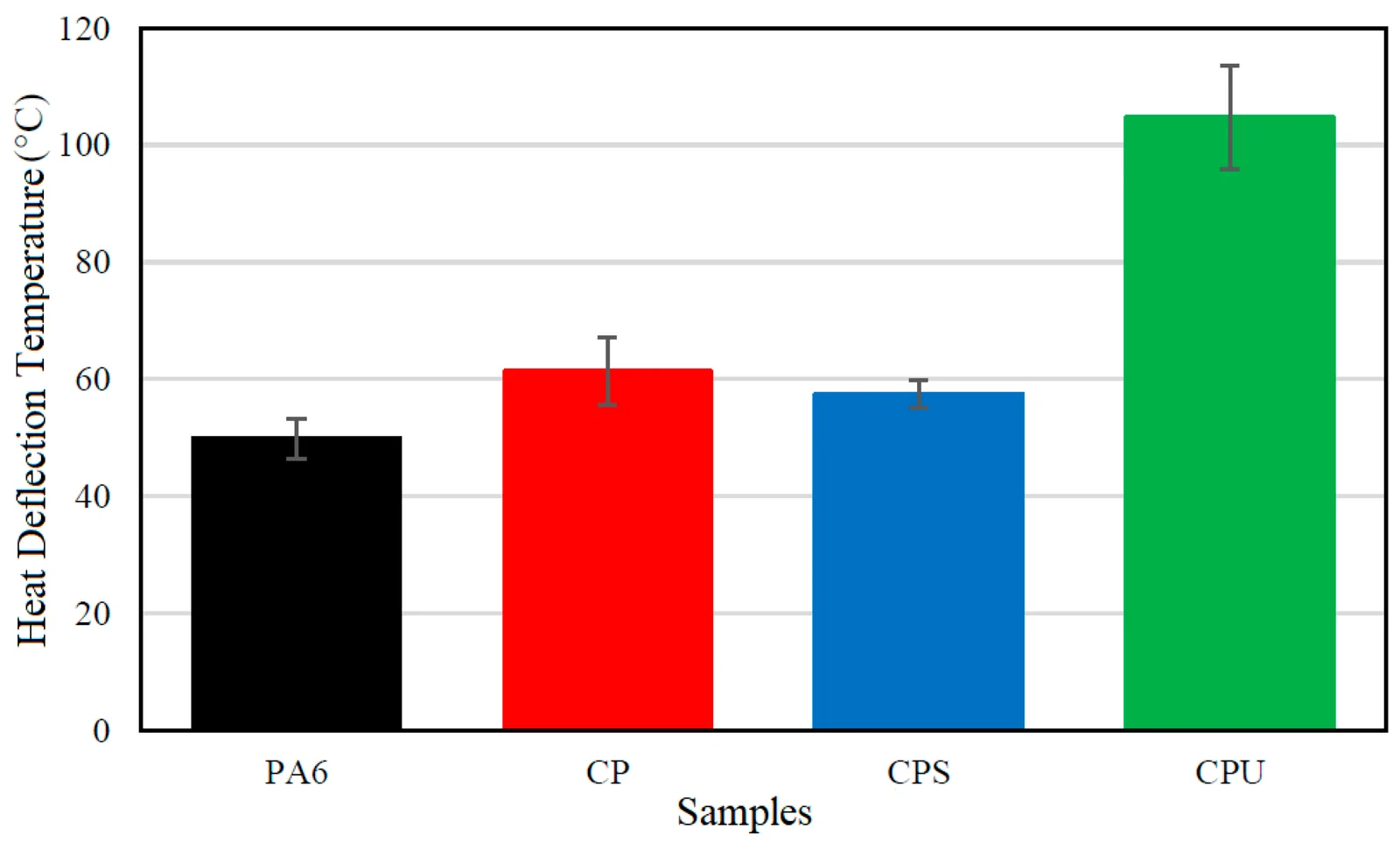
HDT analysis results of the mineral-coated PA6 composites.

Figure 10 shows the heat deflection temperature (HDT) of the composites obtained. Among all the materials, PA6 had the lowest heat deflection temperature (HDT), which indicates that the material loses its mechanical strength at a temperature close to the softening point. This low HDT is primarily due to the high percentage of amorphous regions and the absence of any reinforcing fillers [5].

Cement improved the deformation resistance by the mechanical constraint between the polymer chains. This increase in stiffness leads to better dimensional stability under load at high temperatures [20].

The HDT value of the CPS sample is comparable with the CP sample, but slightly lower (~60 °C). Silica fumes can spread well due to their very fine structure. However, its small particle size may hinder its effectiveness as a mechanical reinforcement. Furthermore, the weak interfacial interaction between the silica fume and the polymer matrix, owing to the surface energy characteristics of the silica fume, could further reduce its reinforcing effect [19].

The CPU sample showed the highest HDT (105 °C), which suggested that the fly ash improved the rigidity of the polymer matrix at higher temperatures. The amorphous silicate structure, high surface area, and stronger interfacial interactions of fly ash [17] are expected to enhance the thermal stability and stiffness of the composite.

#### 3.3.4. The UL94 Vertical Burning Test of the Samples

**Figure 11:**
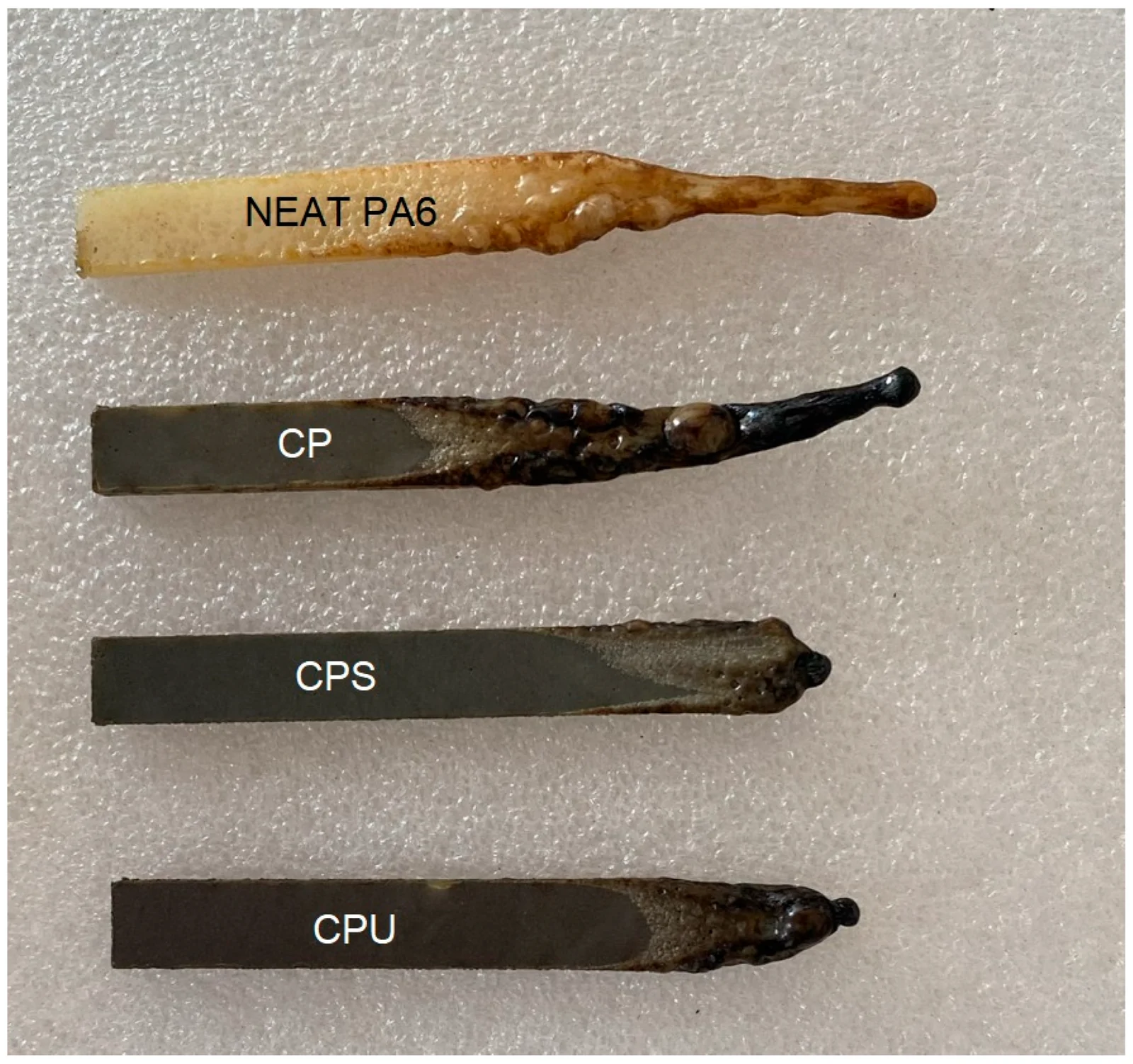
Flammability test samples after the UL-94 vertical burning.

Test results are presented in Table 5 and depicted in Figure 11. The UL 94 vertical burning test results revealed that dripping behavior was observed during combustion. In particular, molten polymer droplets were recorded, and in some cases contributed to the ignition of the cotton indicator placed underneath the specimen. Such behavior indicates that the material does not have enough melt stability when exposed to flame, a critical parameter in the UL 94 classification. Neat PA6 and CP composites achieved a V-2 rating, showing dripping and slower self-extinguishing behavior. CPS was the best performer with a V-0 rating, self-extinguished quickly, and produced no flaming drips due to the barrier effect and char-forming contribution of silica fume. CPU showed a V-1 rating, indicating that the flame resistance was better than that of neat PA6 and CP. This was also confirmed by the mineral content of fly ash, which improves the stability of charring and reduces dripping.

The controlled comparison at a constant total inorganic content revealed that the partial replacement of cement with silica fume or fly ash produced distinct performance profiles rather than a uniform filler effect. The fly ash–cement coating provided the most favorable combination of flexural performance, heat deflection temperature, and residual mass, whereas the silica fume–cement coating was more effective in delaying the onset of thermal degradation, maintaining crystallinity, and improving the UL 94 classification. These findings demonstrate that the performance of mineral-coated PA6 composites is governed by the composition of the coating system and provide a basis for tailoring the mechanical, thermal, and flammability properties of PA6 through pre-extrusion coating.

## 4. Conclusions

The influence of cement and pozzolanic additives, such as fly ash and silica fume, on the mechanical, thermal, and flammability properties of PA6-based composites was investigated within the perspective of this study. These mineral fillers have significantly improved the overall mechanical performance, although the tensile strength did not improve to the same extent. Therefore, further studies should investigate lower silica fume and fly ash contents to identify an optimum loading level that enhances stiffness without substantially reducing tensile and flexural strength.

The heat deflection temperature (HDT) was also significantly improved with the addition of pozzolanic materials, with fly ash providing the most significant improvement, indicating an improved thermal resistance under load. The DSC results indicated that there was no significant change in the melting or crystallization temperature of the polymer matrix upon addition of these additives, suggesting that the crystalline structure of PA6 was not greatly affected.

The results of TGA confirmed the effectiveness of fly ash as a reinforcing and protective filler with the highest residual mass and improved thermal stability. Furthermore, the UL94 flammability tests indicated the improvement of the flame-retardant behavior of PA6 by the mineral additives, and the fire resistance was in the order of silica fume and fly ash.

The results generally indicated that mineral-coated PA6 composites provided a good balance of improved thermal stability, heat resistance, and flame retardancy, and also provided the inherent lightweight benefits of polymeric materials. These properties are well aligned with the growing demand for sustainable and safety-critical materials in the automotive industry. Therefore, mineral-modified PA6 has strong potential to be used as an alternative material as a substitute for traditional plastics. Based on the results of this work, it can be stated that such products could be used without any problems for applications where thermal and fire performance play an important role, such as under the hood and interior structural units.

## Figures and Tables

**Table 1:** Manufacturing design for the hybrid PA6 composites.

Sample ID	Polyamide 6 Polymer (%)	Portland Cement (%)	Silica Fume (%)	Fly Ash (%)
PA6 (Control)	100	-	-	
CP	85	15	-	
CPS	85	10.5	4.5	
CPU	85	10.5	-	4.5

**Table 2:** Burning criteria for UL 94 vertical rating.

Criteria	UL 94 Vertical Rating		
	V-0	V-1	V-2
Duration of combustion for each individual test specimen (s) (subsequent to initial and secondary flame applications)	≤10	≤30	≤30
Duration of combustion and residual glow following the second application of flame (s)	≤30	≤60	≤60
Dripping of ignited specimens (combustion of cotton batting)	No	No	Yes
Combustion to the holding clamp (specimens entirely incinerated)	No	No	No

**Table 3:** TGA results of the mineral-coated PA6 composites.

Sample ID	T5% (°C)	T10% (°C)	Tmax (°C)	Residue (%) at 1000 °C
PA6 (Control)	369.4	396.2	457.6	0.4
CP	371.6	390.8	431.3	11.3
CPS	379.9	398.6	438.5	13.4
CPU	375	395.3	435.7	14.6

**Table 4:** DSC results of the mineral-coated PA6 composites.

Composite ID	Melting		Crystallinity		Xc (%)
	Tm (°C)	ΔHm (J/g)	Tc (°C)	ΔHc (J/g)	
PA6 (Control)	221.6	108.6	186.6	119	47.2
CP	220.8	90.7	186.5	95.9	33.5
CPS	220.9	106.2	188.8	110.4	39.2
CPU	221.9	97.4	189.3	100.2	36

Tm: melting temperature; ΔHm: melting enthalpy; Tc: crystallization temperature; ΔHc: crystallization enthalpy; Xc: degree of crystallinity.

**Table 5:** The UL94 flammability classification of the test samples.

ID	PA6	CP	CPS	CPU
UL Vertical Flammability Class	V-2	V-2	V-0	V-1

## Data Availability

The original contributions presented in this study are included in the article. Further inquiries can be directed to the corresponding author.
